# Supplementary material for: Finding of Novel Galactose Utilizing Halomonas sp. YK44 for Polyhydroxybutyrate (PHB) Production
Source: Polymers (Basel). 2022 Dec 10;14(24):5407. doi: 10.3390/polym14245407 (PMC9782037; doi:10.3390/polym14245407)

Supplementary Table

Table S1. Species identification using 16S rRNA sequencing and antibiotic resistance the identified strain.

Strain	16S rRNA sequencing		Antibiotic resistance				
	Species	Query	Ampicillin	Kanamycin	Gentamycin	Spectinomycin	Chloramphenicol
H. cerina (*Halomonas* sp. YK44)	*H. cerina* SP4	99%	++	-	-	+	-

Table S2. Comparison of GPC analysis result *Halomonas* sp. YK44 film and Authentic PHB film.

	M_n	M_w	PDI
Authentic PHB film	3.19×10^5	4.29×10^5	1.34
H. cerina film	6.90×10^5	7.92×10^5	1.15

Where M_n, number average molecular weight; M_w, weight average molecular weight; PDI, polydispersity index

Supplementary Figure Legends

Figure S1. DSC analysis of PHB from *Halomonas* sp. YK44.

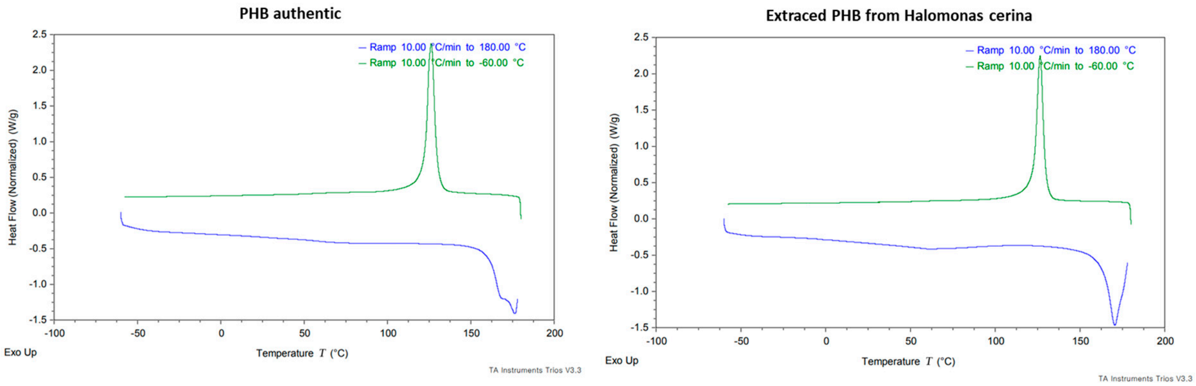

Figure S2. PHB film extracted from *Halomonas* sp. YK44

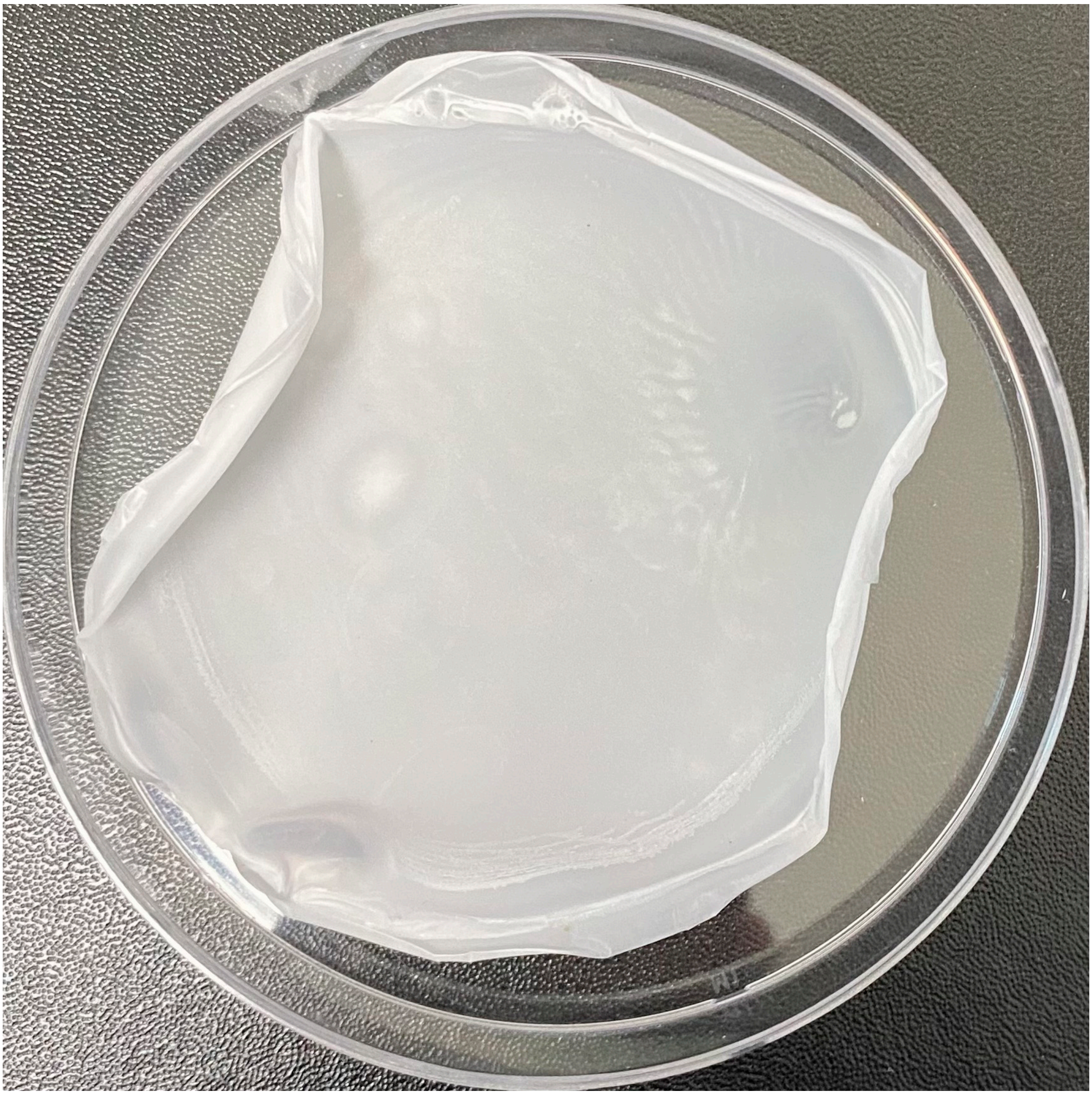

Supplement: Supplementary file 1 [file polymers-14-05407-s001.zip › polymers-2042173-supplementary.pdf]
